# Transactions of the Medical Society of the State of Pennsylvania at Its Annual Session Held in the City of Philadelphia, May, 1852

**Published:** 1852-10

**Authors:** 


*Transactions of the Medical Society of the State of Pennsylvania at its annual session held in the city of Philadelphia, May* 1852. Published by the Society.

The Transactions of the Society for the present year form a very handsome volume of 146 pages, illustrated by three geological charts, all got up in the best style of printing and engraving. The minutes of the Society we have already given very fully in our pages. In addition, this volume contains the Address of the President, Dr. Innes, and various county and committee reports. The former is sensible and well-written, displaying a high ethical tone, and full of sound practical suggestions. Among other topics, Dr. Innes alludes to the seriously defective organization of the National Medical Association, and points out the evils arising from the admission of the representatives of "coteries formed on the *similia similibus* platform." We confidently trust that this grand defect will be speedily remedied, and we agree with Dr. Innes that it can be done effectually only by confining membership in the Association to "delegates exclusively from State and County Medical Societies."

The reports of the County Societies are of very great interest. Several contain a geological and topographical description of the respective counties; and the Schuylkill, Delaware and Lebanon reports are accompanied by geological charts. These, as the Schuylkill County committee suggest, are "not intended as mere temporary matters, but may be found useful for future reference." It will be remembered that the Berks County Society began this work last year, by submitting a geological chart, with remarks as to the prevalence of certain diseases (particularly dysentery,) upon certain formations more than upon others. The frequent references to this opinion in the reports of the present year, show that the professional mind is awakened to the topic, and an extent and industry of observation aroused which must lead to more reliable conclusions. In the report for 1851, the Berks County Society state that the fatal dysentery that prevailed in 1822 was almost exclusively confined to the slate formation, where it still "prevails from time to time, and occasionally proves exceedingly malignant;" and that this disease, "which is so frequently prevalent on all the other formations, never occurs epidemically on the limestone, and even sporadic cases are unfrequent." At the same time they assert that the general standard of health upon the limestone is inferior to that upon the other formations. In the reports for the present year, we have numerous references to this important opinion, and facts cited both *pro* and *con*. The Delaware county report states that "after a careful examination and comparison of facts, the committee are impelled to the belief that the diseases of this county during the year have not been in any manner influenced by geological formation," except, of course, the prevalence of miasmatic disease upon the river alluvium. The Lebanon committee assert that "in the gravel district dysentery prevails more frequently than in other parts of the county, and often in the most malignant form." Dr. Henderson, reporting for Mifflin county, says that the opinion of the Berks county committee is not borne out by his experience, many cases of dysentery occurring "upon the lime rock and among those who use the hard water issuing from it." Dr. Confer, writing from Logan's Valley, Blair County, "principally limestone soil," mentions a violent epidemic dysentery prevailing there in August of last year. Dr. Ogier, of Chester County, describes an epidemic dysentery, commencing on the slate ridge to the south of the limestone valley, and for some time confined to that region, but afterwards extending over the limestone; and adds, that one-third of his patients resided in the valley and used limestone water alone as drink. Dr. Edge, of the same county, noticed that, although one half of his patients resided upon the limestone formation, only five used limestone water as drink, and there were other morbific causes in these cases. He adds that "on this subject there has always prevailed a sentiment in this place, which can be traced back to the experience of Drs. Todd, Kersey and Fairlamb, that the limestone water of our valley had a prophylactic effect against dysentery." Dr. Harry, of the same vicinity, states, as the result of twenty years' observation, that "congestive fever prevailed most in the limestone districts and in the valleys, whereas dysentery prevailed mostly on the hills, and he has never known it to prevail as an epidemic in the limestone district." The Bucks County committee state that the epidemic dysentery which afflicted their county in 1838, was "almost exclusively confined to an elevated sandy ridge," and they "have no recollection of any case originating in the limestone valley immediately adjacent." They admit, however, that this conflicts with the experience of their medical brethren of Montgomery County, in reference to a "dysentery which prevailed in the limestone valley of Plymouth and Whitemarsh in the year 1849." The Berks County committee reiterate the opinion expressed in their last report. They give at length the report of Dr. Bertolet of Oley, upon the dysentery of his neighborhood in the summer of 1851. Dr. B. says that "the districts where dysentery was most common were either of the slate or gravel formations, or slate underlaid with limestone; some few sporadic cases were found in the limestone region, but these could almost invariably be traced to exposure on the gravel."

It will thus be seen that the facts cited are somewhat contradictory, while the weight of evidence seems to be in favor of the comparative immunity of limestone regions from this fatal disease, so prevalent of late years. Another interesting fact appears also—that the disease is generally confined to hot and dry seasons, when the streams are low and many springs and wells nearly dried up. The water from such sources will then contain, as the Lebanon committee suggest, an inordinate proportion of saline matter in solution, and may thus become irritating to the bowels. In some cases, it appears that families drinking water from deep wells, have invariably escaped, while those obtaining their water from open springs, especially with a northern exposure and inadequately shaded, have suffered severely. Another point, which we think has not yet attracted sufficient notice, is the distinction to be drawn between proper epidemic dysentery, such as has devastated whole regions of our State, and that which occasionally prevails in miasmatic districts simultaneously or alternately with fever. Dr. Parrish, of Chester County, describes in the present volume (p. 71,) a dysentery evidently of the latter character. We presume that Dr. Gaston means the same thing when he speaks of the dysentery of his neighborhood as obviously "of septic origin." The same *may* be true of the dysentery of Montgomery County, which, commencing in a peculiarly miasmatic region on the Schuylkill, extended up the limestone valley of Plymouth and Whitemarsh. It is desirable, in future reports, that we should have a statement not only of the topographical relation of the disease to geological structure, but also a notice of any local or particular cause which might operate a deviation from the general law in the case.

The disease which, next to dysentery, appears to have occupied the attention of our country brethren during 1851, is Scarlatina, in regard to which we have several interesting observations and suggestions. The current of opinion in this, as in typhoid fever, sets strongly against any heroic or perturbating treatment. The subordidate importance of intermittent and remittent fevers in these reports, is striking. The case would have been very different twenty years ago. It is true that the counties bordering on the Susquehanna are scarcely represented at all; but we have reports covering the whole valley of the Schuylkill and most of its tributaries to the source. The Lebanon County committee state expressly that "the malarial fevers are rapidly disappearing as the improvements of the country advance."

On the whole, we consider this publication as highly honorable to the Society from which it emanates. We sincerely trust that its next meeting may be still more numerous, and that all the other counties in the State will be roused to action and co-operation.

